# Oral Health Knowledge, Self-Assessed Oral Health Behavior, and Oral Hygiene Practices among the Adult General Population in Croatia

**DOI:** 10.3390/healthcare12010088

**Published:** 2023-12-30

**Authors:** Antonija Tadin, Marija Badrov

**Affiliations:** 1 Department of Restorative Dental Medicine and Endodontics, Study of Dental Medicine, School of Medicine, University of Split, 21000 Split, Croatia; mb91802@mefst.hr; 2 Department of Oral and Maxillofacial Surgery, Clinical Hospital Centre Split, 21000 Split, Croatia

**Keywords:** adults, knowledge, oral behavior, oral health literacy, oral hygiene, practices

## Abstract

Objectives: Emphasizing the significance of optimal oral health for enhancing overall well-being, this study aimed to investigate oral health knowledge, self-assessed oral health behaviors, and oral hygiene practices within the Croatian general population. Materials and Methods: A cross-sectional observational study was conducted online, utilizing a questionnaire collecting sociodemographic characteristics and inquiring about respondents’ oral health knowledge, self-assessed oral health status, oral hygiene habits, and use of oral hygiene products. Collected data underwent both descriptive and regression analyses to unveil patterns and relationships within the dataset. Results: The respondents showed a high level of knowledge about oral health (median score 9, IQR 7.00–10.00, maximum possible score 10), with significant factors for higher knowledge being engaged in dental professions; above-average socioeconomic status; and the use of an electric brush, dental floss, and interdental brushes (*p* ˂ 0.05). Insufficient knowledge, on the other hand, was associated with bleeding gums and daily smoking (*p* ˂ 0.05). Although 69.4% of respondents considered their oral health to be very good, 32.5% reported tooth decay, and 38.2% reported tooth sensitivity. The majority (62.0%) cited regular check-ups as the reason for their last visit to the dentist, with 74.1% feeling no anxiety or discomfort during these visits. Daily toothbrush use was widespread (97.8%), but only 34.1% and 19.1% of respondents reported using floss and/or interdental brushes daily. A remarkable 69.0% of respondents indicated that low oral health literacy and low prioritization contribute to suboptimal oral health. Conclusions: While respondents demonstrated commendable oral health knowledge, the prevalence of reported issues suggests a potential gap between perception and actual oral health status. To address this, targeted educational interventions focusing on comprehensive oral hygiene practices and debunking misconceptions should be prioritized in Croatia.

## 1. Introduction

In contemporary discourse, the paradigm of oral health goes beyond the cosmetic aspect of an esthetically pleasing smile. Beyond the pursuit of white and healthy teeth, oral health is now recognized as a crucial factor in overall well-being. Research findings have consistently emphasized its importance as a fundamental factor in overall quality of life and physical health [1,2].

Impairments to oral health can greatly affect a person’s daily life by causing discomfort and pain and hindering essential functions such as eating, smiling, and speaking properly. Neglecting oral health can promote serious multisystemic diseases such as diabetes and cardiovascular disease [3,4]. Prevalent oral health conditions that pose a significant threat include dental caries, periodontal disease, oral cancer, dental injuries, and tooth loss. Together, these conditions represent a significant global public health problem. Dental caries, for example, is widespread, affecting nearly 100% of the population in many countries, while severe periodontitis affects 5–20% of the adult population [4]. Various factors contribute significantly to an increased risk of oral disease, including inadequate oral hygiene, tobacco use, excessive alcohol consumption, and suboptimal dietary habits. In addition, inadequate oral hygiene can lead to tooth decay, gingivitis, periodontitis, tooth loss, bad breath, and other oral infections caused by bacterial plaque metabolism [5]. Mechanical plaque removal by brushing teeth twice a day with fluoride toothpaste, supplemented by the use of dental floss or interdental brushes, is considered an essential preventive measure for optimal oral hygiene [6,7,8].

Abstaining from tobacco use and moderating alcohol consumption represent additional measures to enhance oral health, mitigating the risk of developing periodontitis, peri-implantitis, xerostomia, as well as certain deleterious conditions such as premalignant lesions and oral cancer. Furthermore, dietary choices characterized by elevated quantities and frequencies of sugary and acidic foods and beverages contribute to an increased susceptibility to dental caries and erosion [9,10]. Adequate hydration is essential to promote adequate saliva production, as saliva plays a central role in preventing the development of dental caries by neutralizing acids and facilitating the removal of food debris [11]. Regular dental visits and check-ups are essential for the maintenance of oral health and the prevention of oral diseases. As some authors have pointed out, a significant proportion of the adult population tends to seek dental care only in emergencies or when in pain, and considers routine dental visits either “unimportant” or “financially prohibitive” [12,13,14]. Studies have shown a clear association between advanced age, lower socioeconomic status, or educational level and self-rated poor oral health [13,14,15,16,17,18].

Several studies conducted in Croatia have examined the oral health and oral hygiene habits of specific demographic groups, including students [19], adolescents [20], soldiers [21], children [22], and pregnant women [23]. The data on the decay–missing–filled (DMF) index in Croatia underlines the persistent problem of dental caries and shows that this is an important public health issue that requires concerted efforts at national and local level. According to data from the Croatian Central Health Information System, the DMF index for children under 12 years of age was recorded at 4.18 between 2013 and 2015, while adults aged 18 to 65 years exhibited a DMF index of 12.5, indicating Croatia’s struggle with a high DMF index [24]. In 2019, the World Health Organization reported a prevalence of untreated caries in permanent teeth among individuals aged 5 years and older of 40.7%, along with a 21.3% prevalence of severe periodontal disease in those aged 15 years and older in Croatia [25]. A 2007 study conducted in the Croatian capital revealed that 12% of nineteen-year-olds had shallow periodontal pockets. Notably, this symptom was significantly prevalent in the 45–54 age group (45.7%) and individuals over 65 (48.2%). The study also emphasized that over 80% of participants required initial periodontal treatment [26]. These findings align with those of another study from 2000, examining the periodontal health of the rural Croatian population. It identified a lack of a healthy periodontium in the over-30 age group in both coastal and mountainous regions. In the coastal regions, 11.76% of young people already had shallow periodontal pockets, while in the mountainous regions, this was observed in only 14.26% from the age of 20. For individuals over the age of 30, over 50% in both regions had either deep or shallow pockets, and approximately 40% of people over the age of 55 in both groups had at least one deep pocket [27]. These elevated indices underscore the urgency of targeted interventions and comprehensive strategies to address and improve the prevalence of dental caries and periodontal diseases in different age groups across the country.

To the best of our knowledge, this study represents pioneering work, as it is the first to include the country’s general population as a study group. The main objective of this study was to investigate (1) oral health knowledge, (2) self-assessed oral health behaviors, and (3) oral hygiene practices in the Croatian general population. The hypotheses underlying this study were based on the assumption that the respondents would have inadequate knowledge and display inappropriate oral hygiene habits. These basic assumptions guided the study, which aimed to shed light on possible gaps in knowledge and practices related to oral health in the study population. Exploring these hypotheses contributes to a more nuanced understanding of the prevailing oral health landscape and can inform targeted interventions to address knowledge gaps and promote improved oral hygiene practices.

## 2. Materials and Methods

This observational cross-sectional study employed convenience sampling and was carried out between December 2022 and February 2023 within the Department of Restorative Dental Medicine and Endodontics at the School of Medicine, University of Split, Croatia. Data collection was facilitated through an online survey administered via a Google Form. Ethical approval for the study was obtained from the Ethics Committee, aligning with established guidelines and regulations, including adherence to the Declaration of Helsinki of the World Medical Association. Participation in the survey was voluntary, and respondents remained anonymous throughout the study.

The study covered the adult general population of Croatia with a total sample of 2126 respondents. The respondents came from all four statistical regions of the Nomenclature of Territorial Units for Statistics (NUTS) in Croatia, namely, the City of Zagreb, Northern, Pannonian, and Adriatic Croatia. The recruitment of respondents was facilitated by the distribution of an online survey link via various social networks. The inclusion criteria required respondents to reside in Croatia, be of legal age, have an email address or be a member of one of the specified social networks, and belong to one of two genders. Minors were intentionally excluded from the study, as were respondents residing outside Croatia and those with missing data.

The sample size for this study was determined utilizing the Sample Size Calculator (Inc. RaoSoft^®^, Seattle, WA, USA), an online tool. The calculation was based on the adult population of the Republic of Croatia as reported in the last census in 2022 (n = 3,223,679) [28]. Employing a 95% confidence level, a population proportion of 50%, and an absolute precision of 5%, the calculated requirement was established at 385 respondents.

The data utilized in this study were obtained through a questionnaire derived from several surveys with a congruent focus [13,14,16,19,20,29,30,31,32,33,34,35]. Comprising 64 questions distributed across seven sections, the questionnaire commenced with the first section (Q1–Q8) gathering sociodemographic data from respondents, such as gender, age, education level, employment status, engagement in dentistry, socioeconomic status, place of residence, and its population. The second section (Q9–Q18) was dedicated to probing oral health knowledge, with respondents selecting from three options: “Yes”, “No”, or “I don’t know”. In this study, a scoring system was introduced in which a correct answer (“Yes”) was given a score of one, while an incorrect answer was given a score of zero. The cumulative score of correct answers for each respondent was then calculated based on the number of correct answers and served as a quantitative measure for assessing the individual’s level of knowledge. Utilizing Bloom’s cutoff values, respondents were categorically classified based on their overall knowledge scores in the study. Those scoring between 80 and 100% (8 to 10 points) were characterized as possessing “good knowledge”, while individuals scoring between 60 and 79% (6 to 7 points) were designated as having “moderate knowledge”. Conversely, respondents scoring below 60% (0 to 5 points) were identified as having “poor knowledge” within the context of this study [36]. The third section (Q19–Q30) encompassed questions pertaining to the utilization of dental services. This included inquiries about the type of dental care received, the timing and reasons behind the last dental visit, experiences with dental anxiety or phobia, self-assessment of oral health and general health status, knowledge about oral health and hygiene, sources of oral health information, attitudes toward the importance of oral health, interest in oral health education, and reasons for poor oral health. The fourth section (Q31–Q44) detailed self-reported oral issues and treatments received by respondents, encompassing aspects like tooth decay, dental fillings, extracted teeth, bleeding gums, dentin hypersensitivity, etc. The fifth section (Q45–Q51) focused on examining the correlation between the frequency of everyday use of oral hygiene aids, including toothbrush, dental floss, interdental brushes, tongue scrapers, toothpaste, and mouthwash. The sixth section (Q52–Q59) comprised questions concerning toothbrushing practices and habits. These included inquiries about preferred toothbrush hardness, intervals for replacing a toothbrush, type of toothbrush used, duration of toothbrushing, frequency of rinsing while brushing, approximate amount of toothpaste used, and a question related to fluoride concentration in toothpaste. The final section, the seventh, included five questions (Q60–64) relating to respondents’ daily dietary and lifestyle habits, covering aspects such as smoking, alcohol and coffee consumption, and the consumption of sweet drinks and snacks.

The survey instrument was administered in the Croatian language; however, for the study’s objectives, the questions underwent translation both from English to Croatian and vice versa. Prior to the formal implementation of the study, a pilot test of the questionnaire was conducted with a cohort of 50 students and employees affiliated with the School of Medicine, University of Split. This pilot test aimed to assess the questionnaire’s clarity, readability, and overall comprehensibility. It is noteworthy that individuals who participated in the pilot study were subsequently excluded from the final sample. Furthermore, an assessment of the questionnaires’ reliability was conducted, revealing a Cronbach’s alpha value of 0.687 for questions related to oral health knowledge.

The normality of data distribution was assessed using the Kolmogorov–Smirnov test. Descriptive statistics were employed for data analysis; categorical variables are presented as frequencies and percentages, and quantitative variables as means with standard deviations or medians with interquartile ranges. To discern associations between oral health knowledge values and sociodemographic factors as well between respondents self-assessed oral health status, everyday oral health practices and lifestyle habits, a generalized linear model analysis was conducted. Statistical significance was established at *p* < 0.05. The Statistical Package for the Social Sciences, version 26 (SPSS, IBM Corp, Armonk, NY, USA), was utilized for data analysis.

## 3. Results

Table 1 provides an overview of the socioeconomic attributes of the study respondents. A total of 2126 adult respondents actively contributed to the research, with women constituting a substantial majority at 79.9%. The median age of the respondents was 25, with an interquartile range (IQR) spanning from 22 to 39. The age range observed in the study varied from a minimum of 18 years to a maximum of 75 years.

The median knowledge score among respondents pertaining to oral health was 9, with an interquartile range (IQR) of 7.00–10.00, with a maximum attainable score of 10 (ranging from a minimum score of 0 to a maximum of 10). Noteworthy findings indicate that 56.2% of the respondents exhibited a knowledge score equivalent to or exceeding the median level. There were no statistically significant variations in oral health knowledge observed among respondents based on sex, age, or education level. However, certain demographic subgroups demonstrated notably heightened levels of knowledge regarding oral health. Specifically, students, individuals employed within the field of dentistry, those with above average socioeconomic status, and residents of North and Adriatic Croatia exhibited significantly superior knowledge of oral health matters (*p* < 0.05).

Table 2 illustrates the distribution of correct responses (“Yes”) to statements pertaining to oral health and oral hygiene knowledge. A noteworthy majority of respondents, more than 80%, accurately answered 7 out of 10 statements. However, awareness regarding the impact of alcohol consumption on oral health was comparatively lower, with only 66.9% providing correct responses. Additionally, a substantial percentage of respondents, 34.3%, lacked knowledge about the effects of fluoride on oral health. Only 20.4% of respondents expressed disbelief in the recommendation that teeth should be brushed twice a day for at least two minutes.

Table 3 summarizes questions related to the usage of dental services and self-assessment of oral health and oral hygiene status and knowledge. The majority of the respondents (67.5%) used public dental services. More than half (54.9%) of them had visited their dentist less than 6 months ago, the reason for which was mainly a regular check-up (62%). An emergency was the reason for 10.1% of them; 21.5% of respondents stated that they experience moderate anxiety while visiting the dentist, and 4.4% of them had dental phobia. Most of them assessed their oral health (69.4%) as “very good. Surprisingly, 40.8% of them assessed their oral health knowledge as “moderate”. Oral hygiene knowledge was rated as “very good” by 67.1% of the respondents. The dentist was a main source of oral health information for most of the respondents (74.6%). A great majority (96.1%) of them considered oral health and oral hygiene knowledge as very important, but, surprisingly, only 59.4% of them showed interest in oral health education. According to their opinion, the main reason for suboptimal oral health and oral hygiene in Croatia seem to be poor oral health literacy and low oral health prioritization.

Table 4 outlines the prevalence of self-reported conditions and oral cavity treatments among the respondents. Common issues included tooth decay (32.5%) and dentin hypersensitivity (38.2%). Over 90% had dental fillings, and approximately half had underwent tooth extraction (44.2%) or endodontic treatment (44.7%). Individuals experiencing bleeding gums exhibited lower oral health knowledge (*p* = 0.032), while those with a burning mouth demonstrated significantly better oral health knowledge (*p* = 0.022).

Table 5 illustrates the frequency of daily oral hygiene aid usage. The majority of respondents (88.8%) used a manual toothbrush daily, and nearly all (96.9%) used toothpaste. However, interdental care was lacking, with only 34.1% using dental floss and 19.1% using interdental brushes daily. Daily use of toothbrush, dental floss, interdental brushes, and toothpaste was associated with a higher score for oral health knowledge (*p* < 0.05).

Table 6 provides a comprehensive overview of the respondents’ use of oral hygiene products and practices. It is noteworthy that 41.3% of the respondents preferred medium–hard brush bristles, while 38.2% used soft bristles. The majority (64.0%) changed their toothbrush within 3 months; 18.6% did so after 3 months or longer. Manual toothbrushes were predominantly used (91.0%), and most respondents (72.9%) brushed for 2 to 3 min. In terms of rinsing habits, 54.4% rinsed their toothbrush during brushing once or twice and 45.3% rinsed their mouth thoroughly after brushing. About 35.7% used an average amount of toothpaste of 1 cm, while the majority (56.8%) used a smaller amount. Surprisingly, 80.0% did not know the fluoride concentration in their toothpaste, and 3.8% used fluoride-free toothpaste.

Table 7 shows the daily lifestyle and dietary habits of the respondents. A quarter of them were smokers, and the study revealed that they knew less about oral health compared to the nonsmokers (*p* = 0.005). In addition, 18.4% of them consumed sugary drinks daily, and 13.8% ate sweet snacks with meals.

## 4. Discussion

Recognizing that sound oral health knowledge and positive habits have a significant impact on oral health and overall health and quality of life [1,2], this study aimed to determine the correlation between the knowledge, practice, and reported oral health status of Croatian adult population. The results showed that the respondents had a commendable knowledge of oral health, but this did not match their reported daily oral hygiene habits or self-assessed oral health status. It is noteworthy that individuals with a higher socioeconomic status, particularly students and employees, especially in the dental field, had better oral health knowledge. A higher socioeconomic status is often associated with a deeper understanding of oral health. This may stem from access to better educational resources and greater availability of preventive dental care, collectively contributing to their heightened awareness of the importance of oral health [19,37,38]. As expected, employees in dental medicine often possess advanced knowledge of oral health due to specialized education and professional experience in the field of dentistry. Their daily environment and work practice provide them with direct access to information and experiences that enhance their understanding of oral health [19,29].

Ten questions were used to assess the respondents’ general knowledge of oral health, and they showed a high level of oral health knowledge (median score 9, IQR 7.00–10.00, maximum possible score 10). The respondents answered almost all questions correctly with a high degree of confidence (between 60 and 90%). A total of 38.5% of respondents answered all questions correctly, while only 0.4% did not give a single correct answer. The majority of respondents in this study exhibited a high comprehension of oral health and acknowledged its significant correlation with an individual’s quality of life. This discovery aligns with analogous outcomes found in studies conducted among student populations in Split-Dalmatia County, Croatia, and among water polo players [19,39]. Furthermore, parallels can be drawn with a comparative study involving adolescents from Portugal, Sweden, and Romania [33], as well as a study involving Polish patients with periodontitis [40]. In addition, slightly more than half of the respondents were aware of the preventive role of fluoride in dental caries. They knew that fluoride helps to prevent damage to the tooth surface, facilitate its remineralization, and inhibit bacterial growth. These findings are similar to the results of a study conducted on a student population in Saudi Arabia [29]. Conversely, a study of oral health behavior and attitudes of adults in Lithuania showed that more than a half of the respondents did not show an understanding of any effect of fluoride on oral health [41].

For optimal oral health, it is crucial to schedule regular visits to the dentist. Individuals who are routine dental visitors are associated with fewer missing teeth and tooth decay [42]. As shown in this study, slightly more than half of the respondents (54.9%) had visited their dentist in less than 6 months, of which 62% of them had scheduled a regular check-up appointment. On the other hand, only 10.1% had their last dental visit due to an emergency. Some studies showed extremely higher proportion of patients who only sought dental care only when in pain or emergency, such as studies among adults from Lithuania [41], South America [43], and Poland [16]. The majority of respondents in this study (69.4%) reported perceiving their oral health as “very good”. A slightly higher proportion of individuals self-reporting “very good” oral health has been found in a self-reported oral health study from Germany [15] and in a British cohort study [18]. However, an even larger percentage (78.3%) in this study indicated that they considered their general health as “very good”. This self-perception of general health being better than oral health was also established by Norwegian study conducted on their adult population [12]. Dental professionals have a responsibility to promote oral health behavior and give proper information on practicing oral hygiene [44]. In this study, the dentist was the main source of oral health and oral hygiene information to 74.6% of the respondents. This finding correlates with that of the study of students from the University of Split [19]. A comparative study of adolescents from Portugal, Romania, and Sweden found that the dentist was the main source of oral health information to a somewhat smaller percent (53.6%) of their respondents [33]. When queried about the primary factors contributing to suboptimal oral health and hygiene in Croatia, a majority (69%) identified insufficient oral health literacy and a lack of emphasis on oral health as the main reasons. Some other studies had pointed out that the main reason for irregular dental visits and insufficient oral health among their population was the cost of dental services [12,14]. However, slightly less than half of the respondents (45.2%) in this study also mentioned this issue. Interestingly, a great majority of the respondents (96.1%) perceived oral health knowledge as “very important”, but 40.6% of them did not show interest in receiving professional oral health and oral hygiene education. A lack of interest in oral health, as well as going regularly to the dentist, was noted in studies from Norway [12] and Portugal [14].

The DMF index is most effective tool used in Croatia to determine oral health status. According to research from 2015 conducted in Croatia, the average DMF index of Croatian adult population was 12.5, which happened to be one of the highest in Europe [24]. Furthermore, the most common reported oral health problems in this study were dental fillings (90.1%), root canal treatment (44.7%), tooth loss (44.2%), tooth decay (32.5%), and dentin hypersensitivity (38.2%). A study of dental patients in China revealed that their participants reported having dental caries (17.4%) and missing teeth (10.3%) less frequently than Croatian population in this study [45]. Gum bleeding is among the indicative symptom of gingivitis and periodontal disease [40]. In this study, one-fifth (20.7%) of the respondents reported on having this issue. Additionally, individuals experiencing bleeding gums exhibited statistically lower oral health knowledge (*p* = 0.032). This concern was already emphasized in a study conducted among periodontal patients, confirming that low oral health literacy is closely linked to the development of gingivitis and periodontitis, with a higher likelihood of experiencing a more severe disease course [40]. Additional studies have documented a higher incidence of self-reported issues with gingival bleeding, with examples including over half of the participants in Polish [16] and South American studies [43]. A study concentrating on the oral health of the adult population in China recorded that slightly over half (50.5%) of the recent dental visits were attributed to gingival bleeding [45]. While only a small proportion (2.1%) of the participants in this study reported experiencing burning mouth syndrome, they showed statistically significant better understanding of oral health knowledge (*p* = 0.022). The unpredictability of this disease may account for the observed phenomenon, as patients actively seek assistance and educate themselves on practicing optimal oral hygiene [46].

Ensuring consistent adherence to proper oral hygiene is crucial in preventing dental caries, gum inflammation, periodontitis, and other oral diseases. Therefore, an effective oral hygiene entails brushing teeth thoroughly twice a day, dedicating 2–3 min to each session [6]. This study shows that over 90% of the Croatian population is aware of the importance of proper oral hygiene and its role in preventing the most common oral diseases. Accordingly, 97.8% brush their teeth with a toothbrush, 88.8% of respondents brush their teeth daily with a manual toothbrush, while 19.8% of them brush daily with an electric toothbrush. Similar daily toothbrushing habits have also been established in other self-reported oral hygiene behaviors studies, including studies among Polish [16], Chinese [45], Italian [47], and Danish [48] adults. Interdental hygiene is crucial for maintaining optimal oral health by addressing areas that may be overlooked by regular brushing alone [8]. It has been confirmed that individuals who perform interdental cleaning are less likely to suffer from coronal caries, interproximal coronal caries, missing teeth, and periodontitis compared to nonusers of these tools. Furthermore, a positive correlation was found between a higher frequency of interdental cleaning and improved periodontal health as well as improved tooth structure integrity [49]. Although 84.3% of the respondents in this study confirmed that dental floss and interdental brushes should be used every day as a proper oral hygiene measurement, the subjects demonstrated unsatisfactory interdental hygiene. It is important to emphasize that 61.3% of respondents did not use interdental hygiene products. Furthermore, only 34.1% of them used dental floss, and only 19.1% cleaned with interdental brushes daily. This lack in interdental hygiene was also present in other studies based in Poland [16], Italy [47], and Denmark [48]. However, the findings of a study involving adult patients with periodontal disease revealed a notably higher prevalence of daily dental floss (64%) and interdental brush (26%) usage. This satisfactory oral hygiene behavior among this specific population could be attributed to the influence of dentists [40]. This study further demonstrated a substantial correlation between the regular use of interdental aids and a heightened level of oral health knowledge (*p* < 0.05). This finding was also present in a study conducted among students at the University of Split [19].

Most respondents in this study used a manual toothbrush (91%), either medium or soft, to maintain their oral hygiene. The manual toothbrush was mainly used by students in Croatia [19] and by orthodontic patients in China and New Zealand [50], and by a smaller percentage of the population in studies from Bucharest [31] and Bialystok [40]. In this study, we neither asked the reasons for the respondents’ decision to brush their teeth manually or electrically nor did we investigate the correlation between the occurrence of gingivitis and caries and the type of toothbrush used. Investigating such aspects would be interesting and useful. Previously published research has confirmed that there is no discernible advantage of daily electric toothbrushing over daily manual toothbrushing in terms of oral hygiene or clinical parameters when the manual toothbrush is used correctly [51]. Numerous factors influence plaque removal, such as the design of the toothbrush, brushing method, your individual ability, the frequency and duration of brushing, and the properties of the bristles. The choice of bristle type is crucial as it is in direct contact with the teeth and gums and affects gum health, dentin sensitivity, soft tissue trauma, and plaque control. Hard bristle toothbrushes in particular cause more gum lesions than medium and soft bristle brushes [52,53]. A previous study conducted on a student population confirmed the preference for medium or soft toothbrushes in daily oral hygiene among Croatians [19].

This study showed satisfactory oral hygiene behavior, with 72.9% of respondents brushing their teeth for two to three minutes and 64% of them changing their toothbrush every three months. This optimal duration of tooth brushing was achieved by slightly more than half (51%) of the caries-active Swedish adolescents [35] and by 70.5% of the students from Split, who had a lower frequency of toothbrush changes after three months [19]. A large majority (96.9%) of respondents in this study used toothpaste every day. Daily brushing with toothpaste was statistically associated with higher oral health knowledge (*p* = 0.045). This correlates with the results of studies among students from Split, Croatia [19] and Jeddah, Saudi Arabia [29]. Fluoride has the ability to prevent tooth decay by remineralizing the enamel and making the teeth more resistant to acid attacks by bacteria. In addition to brushing your teeth, it is recommended to use toothpaste with a fluoride concentration of 1000–1500 ppm [7]. In this study, 34.3% of the population showed no understanding of the effects of fluoride on oral health, while 80% of them did not know how many ppm of fluoride their toothpaste contained. Despite the widespread use of fluoride toothpaste as a standard measure to prevent tooth decay, a notable finding is that many respondents were unaware of the fluoride concentration in the toothpaste they used. For example, a study in Ethiopia and China found that half of the respondents did not know whether they used fluoridated toothpaste [54,55]. To prolong the fluoride effect, it is important not to rinse the toothbrush with water while brushing and not to rinse the mouth immediately after brushing [56]. Current recommendations suggest that rinsing with water after brushing with fluoride toothpaste should be minimal or avoided as it may reduce the benefits of fluoride [57]. A total of 48.1% of the respondents in this study rinsed lightly with water after brushing, while 45.3% of them rinsed extensively, which is consistent with similar results from a study conducted in Sweden in different age groups [58]. This habit was also found in caries-active adolescents in Sweden [35]. For adults, it is generally recommended to use a pea-sized amount of toothpaste to ensure effective fluoridation [58]. The majority of study respondents reported using approximately 1 cm of toothpaste on a regular toothbrush (35.7%), a practice consistent with the results from Sweden [58]. Although the generally accepted amount of toothpaste is considered sufficient for effective tooth cleaning (pea-sized), recent studies suggest that larger amounts of toothpaste result in a significantly higher cleaning effect [59].

An unhealthy diet, tobacco consumption, harmful alcohol consumption, and poor oral hygiene are considered risk factors for oral diseases [60]. About 25% of the study participants identified as smokers, which is a slightly lower prevalence compared to the results of a study conducted in Croatia [61]. Smokers showed lower oral health knowledge than nonsmokers, a trend also observed in studies from Oman and Germany [62,63]. A balanced diet and limiting sugar consumption are important practices that contribute to the prevention of caries and periodontal disease. Of those surveyed, 18.4% consumed sugary drinks daily, and 13.8% ate sweet snacks with meals. While the link between a high-sugar diet, alcohol consumption, and impaired oral health has been established, a clear link with insufficient knowledge about oral health has yet to be proven [64,65].

This study is subject to several limitations arising from its conception. The cross-sectional nature of the study precludes the identification of cause–effect relationships. As the survey was based on self-assessment without clinical dental examinations, an objective assessment of the respondents’ oral health status was not feasible. The use of an online survey leads to a selection bias, limiting participation to people with internet access and possibly excluding parts of the elderly and rural population. The sample size, sampling method, and cross-sectional design of the study may not accurately reflect the wider population. In particular, the over-representation of younger adults, women, and residents of Adriatic Croatia leads to potential biases, suggesting that the results could be different if the population was more diverse. A methodological limitation arises from the random sampling approach, which relies on the availability and voluntary consent of respondents. The closed-ended questionnaire used in the study, while practical, may not capture all nuanced aspects of respondents’ knowledge, attitudes and practices. To gain a more comprehensive understanding, a qualitative approach is recommended for future research. A qualitative study would address the barriers, experiences, and reasons that contribute to suboptimal implementation of oral health practices, allowing for a more comprehensive exploration of the topic. Future research could benefit from investigating the relationship between oral hygiene habits, and the occurrence of noncarious lesions and gingival recession. In addition, investigating the interplay between tooth brushing techniques, the use of interdental aids, and the incidence of periodontal disease and caries could also provide valuable insights.

Future research efforts should look more closely at the socioeconomic factors that influence oral health knowledge and practices in the adult population. Longitudinal studies that track changes in oral health behaviors over time can provide a dynamic understanding of evolving practices. To increase the reliability of the results, future studies should not only include self-reported measures of oral health but also validate this information through clinical examination. In addition, an assessment of the correct implementation of oral hygiene measures would further increase the completeness of the research findings.

## 5. Conclusions

This study provides valuable insights into the knowledge, self-reported practices, and state of oral health among adult patients in Croatia. The respondents demonstrated high levels of oral health knowledge, a factor that may correlate with higher-than-average socioeconomic status, thereby identifying potential areas for targeted education campaigns. The results on oral hygiene practices indicate commendable adherence to daily tooth brushing and toothpaste use. However, the limited use of interdental care products suggests the value of educational campaigns emphasizing the importance of comprehensive oral hygiene practices. The prevalence of self-reported conditions, particularly dental caries and dentine hypersensitivity, underscores the importance of addressing these common oral health problems as part of public health initiatives. Of note, a significant percentage of respondents were unaware of the fluoride concentration in their toothpaste, indicating a potential gap in oral health literacy. This highlights the importance of raising awareness of the ingredients of oral hygiene products, including fluoride content. In conclusion, this study contributes to our understanding of the oral health dynamics among adult patients in Croatia. The patterns of knowledge and behaviors identified highlight the need for targeted interventions to address specific gaps and ultimately improve the oral health of the population.

## Figures and Tables

**Table 1:** Generalized linear model analysis of predictors (sociodemographic and professional characteristics) for a higher total oral health knowledge score.

Characteristic		n (%)	OR (95% CI)	*p*-Values
**Sex**	Man	427 (20.1)	Reference	
	Woman	1699 (79.9)	1.116 (0.891–1.397)	0.341
**Age (years)**	≤30	1309 (61.6)	Reference	
	31–44	537 (25.3)	−0.120 (−0.379–0.140)	0.365
	45–59	207 (9.7)	−0.095 (–0.441–0.250)	0.589
	≥60	73 (3.4)	0.323 (−0.428–1.2073)	0.400
**Education level**	Elementary school	15 (0.8)	Reference	
	High school	1147 (53.9)	1.125 (−0.146–2.396)	0.032
	Bachelor’s degree	373 (17.5)	1.274 (−0.008–2.555)	0.350
	Master’s degree	533 (25.1)	1.134 (−0.143–2.410)	0.531
	M.Sc./Ph.D.	58 (2.7)	0.820 (−0.554–2.194)	0.274
**Employment**	Unemployed	150 (7.1)	Reference	
	Employed	1019 (47.9)	0.386 (0.340–0.737)	0.032 *
	Student	880 (41.4)	0.406 (0.410–0.771)	0.029 *
	Retired	77 (3.6)	−0.060 (−0.829–0.709)	0.879
**Working in dentistry**	No	1999 (94.0)	Reference	
	Yes	127 (6.0)	3.283 (2.283–4.282)	≤0.001 *
**Socio–economic status**	Below average	142 (6.7)	Reference	
	Average	1650 (77.6)	0.101 (−0.234–0.452)	0.534
	Above average	334 (15.7)	0.710 (0.308–1.111)	0.001 *
**Region of Croatia**	City of Zagreb	356 (16.8)	Reference	
	Adriatic Croatia	1248 (58.7)	0.320 (0.079–0.561)	0.009 *
	North Croatia	249 (11.7)	0.386 (0.380–0.735)	0.030 *
	Pannonian Croatia	273 (12.8)	0.146 (−0.184–0.475)	0.387
**Place of living**	Rural	752 (35.4)	Reference	
	Urban	1374 (64.6)	0.173 (−0.021–0.367)	0.081

Data are presented as numbers (percentages). Reference knowledge category is “low”. OR, odds ratio; 95% CI, 95% confidence interval. * *p* ≤ 0.05.

**Table 2:** Frequency of correctly answered questions on oral health knowledge.

Statement	n (%)
**Oral health is closely related to an individual’s quality of life. (“Yes”)**	1918 (90.2)
**The most common oral diseases are dental caries, periodontitis, and oral cancer. (“Yes”)**	1798 (84.6)
**Diet affects the development of dental caries, periodontitis, and oral cancer. (“Yes”)**	1900 (89.4)
**Smoking is associated with the occurrence of oral cancer and periodontitis. (“Yes)**	1855 (87.3)
**High alcohol intake is associated with an increased risk of developing oral cancer, periodontitis, and dental caries. (“Yes”)**	1422 (66.9)
**Poor oral hygiene can lead to the development of dental caries and periodontitis. (“Yes”)**	2069 (97.3)
**Proper oral hygiene can prevent dental caries and periodontitis. (“Yes”)**	1992 (93.7)
**Teeth should be brushed two times a day for at least two minutes with fluoride toothpaste. (“Yes”)**	1693 (79.6)
**Fluorides prevent dental caries by preventing damage of tooth surface, helping its remineralization and preventing bacterial growth. (“Yes”)**	1397 (65.7)
**As a proper oral hygiene measurement, dental floss, interdental brushes and tongue scraper should be used every day. (“Yes”)**	1793 (84.3)
**Data are presented as numbers (percentages).**	

**Table 3:** Habits of respondents related to the usage of dental health services and self-perceived knowledge of oral health.

Question	Answer	n (%)
**Type of dental healthcare service utilized**	Public dental services	1434 (67.5)
	Private dental services	692 (32.5)
**Time elapsed since the last dental visit**	Less than 6 months	1168 (54.9)
	Less than a year	418 (19.7)
	1–2 years	366 (17.2)
	3 years and more	174 (8.2)
**Reason of the last dental visit**	Emergency	214 (10.1)
	Elective dental treatment	452 (21.3)
	Regular check-up	1319 (62.0)
	Do not remember	146 (6.6)
**Attitude toward dental visits**	No anxiety or discomfort	1574 (74.1)
	Anxiety and discomfort	458 (21.5)
	Dental phobia	94 (4.4)
**Self-rated oral health**	Very good	1476 (69.4)
	Fair	518 (24.4)
	Poor	132 (6.2)
**Self-rated general health**	Very good	1665 (78.3)
	Fair	404 (19)
	Poor	57 (2.7)
**Self-rated oral health knowledge**	Very good	1129 (53.1)
	Fair	867 (40.8)
	Poor	130 (6.1)
**Self-rated oral hygiene knowledge**	Very good	1426 (67.1)
	Fair	655 (30.8)
	Poor	45 (2.1)
Main source of oral health and oral hygiene information *	Family	1016 (47.8)
	Dentist	1587 (74.6)
	Media	833 (39.2)
	School	771 (36.3)
	Other	232 (10.9)
**Importance of oral health and oral hygiene knowledge**	Very important	2043 (96.1)
	Moderately important	75 (3.5)
	Non important	8 (0.4)
**Interest in oral health and oral hygiene education**	Yes	1263 (59.4)
	No	382 (18.0)
	Do not know	481 (22.6)
The main reason of poor oral health and oral hygiene in Croatia *	Price	961 (45.2)
	Availability	444 (20.9)
	Poor oral health literacy	1470 (69.1)
	Low oral health priority	1469 (69.0)
	Lack of preventive programs	846 (39.8)
	Other	61 (2.9)

Data are presented as numbers (percentages). * Multiple answers possible.

**Table 4:** Assessment of the relationship between self-reported oral health status (“yes”/”no”) and oral health knowledge using a generalized linear model (GLM) analysis.

Oral Health Status	n (%)	Knowledge Score	
		OR (95% CI)	*p*-Values
**Tooth decay (“Yes”)**	691 (32.5)	0.154 (−0.347–0.038)	0.117
**Dental filling (“Yes”)**	1916 (90.1)	0.232 (−0.067–0.530)	0.128
**Root canal treatment (“Yes”)**	951 (44.7)	0.064 (−0.123–0.251)	0.530
**Extracted tooth (“Yes”)**	939 (44.2)	−0.110 (−0.293–0.074)	0.243
**Bad breath/halitosis (“Yes”)**	289 (13.6)	−0.244 (−0.510–0.022)	0.073
**Gum inflammation (“Yes”)**	295 (13.9)	−0.021 (−0.299–0.256)	0.880
**Bleeding gums (“Yes”)**	441 (20.7)	−0.256 (−0.490–0.022)	0.032 *
**Mouth ulcers (“Yes”)**	139 (6.5)	0.105 (−0.257–0.468)	0.569
**Burning mouth (“Yes”)**	45 (2.1)	0.796 (0.116–1.447)	0.022 *
**Dentin hypersensitivity (“Yes”)**	812 (38.2)	0.066 (−0.119–0.252)	0.484
**Orthodontic appliance: fixed and aligners (“Yes”)**	226 (10.6)	0.259 (−0.256–1.630)	0.180
**Dental implant (“Yes”)**	147 (6.9)	0.099 (−0.452–0.255)	0.584
**Dentures (“Yes”)**	73 (3.4)	−0.018 (−0.468–0.505)	0.942
**Crowns, bridges, veneers (“Yes”)**	344 (16.2)	−0.141 (−0.393–0.111)	0.273

Data are presented as numbers (percentages). Reference knowledge category is “low” and “no”. OR, odds ratio; 95% CI, 95% confidence interval. * *p* ≤ 0.05.

**Table 5:** Assessment of the relationship between self-reported daily use of oral hygiene aids among respondents (“yes”/”no”) and oral health knowledge using a generalized linear model (GLM) analysis.

Oral Hygiene Aids	n (%)	Knowledge Score	*p*-Values
		OR (95% CI)	
**Manual toothbrush (“Yes”)**	1887 (88.8)	−0.010 (−0.404–0.384)	0.962
**Electric toothbrush (“Yes”)**	422 (19.8)	0.460 (0.116–0.803)	0.009 *
**Dental floss (“Yes”)**	724 (34.1)	0.370 (0.154–0.586)	0.001 *
**Interdental brushes (“Yes”)**	406 (19.1)	0.391 (0.104–0.678)	0.008 *
**Tongue scraper (“Yes”)**	386 (18.2)	0.231 (−0.035–0.497)	0.089
**Toothpaste (“Yes”)**	2060 (96.9)	0.552 (0.013–1.092)	0.045 *
**Mouth wash (“Yes”)**	730 (34.3)	0.019 (−0.205–0.243)	0.866

Data are presented as numbers (percentages). Reference knowledge category is “low” and “no”. OR, odds ratio; 95% CI, 95% confidence interval. * *p* ≤ 0.05.

**Table 6:** Tooth brushing habits among respondents.

Characteristics		n (%)
**Type of toothbrush** *	Manual toothbrush	1933 (91.0)
	Electric rotary—oscillating	303 (14.3)
	Electric sonic—vibrating	168 (8.0)
**Hardness of the toothbrush fibers**	Ultra soft	331 (15.6)
	Soft	812 (38.2)
	Medium	877 (41.3)
	Hard	106 (4.9)
**Frequency of toothbrush change**	Every month	370 (17.4)
	Up to three months	1361 (64.0)
	More than three months	395 (18.6)
**Duration of brushing**	Less than a minute	308 (14.4)
	2–3 min	1549 (72.9)
	More than 3 min	269 (12.7)
**Fluoride concentration in toothpaste**	Do not know	1701 (80.0)
	500 ppm	98 (4.6)
	1000 ppm	102 (4.8)
	1500 ppm	145 (6.8)
	Fluoride-free	80 (3.8)
**Amount of used toothpaste**	Pea-sized	577 (27.1)
	0.5 cm	631 (29.7)
	1 cm	758 (35.7)
	2 cm	160 (7.5)
**Toothbrush rinsing habits during brushing**	Never	665 (31.3)
	Once or twice	1158 (54.4)
	More than twice	303 (14.3)
**Postbrushing rinsing behavior**	Spitting without rinsing	141 (6.6)
	Rinsing slightly	1022 (48.1)
	Rinsing extensively	963 (45.3)

Data are presented as numbers (percentages). Abbreviations: ppm, parts per million. * Multiple answers possible.

**Table 7:** Assessment of the relationship between self-reported daily eating and lifestyle habits (“yes”/”no”) and oral health knowledge using a generalized linear model (GLM) analysis.

Eating and Lifestyle Habits	n (%)	Knowledge Score	*p*-Values
		OR (95% CI)	
**Smoking (“Yes”)**	546 (25.7)	−0.289 (−0.492–0.086)	0.005 *
**Alcohol consumption (“Yes”)**	29 (1.4)	−0.083 (−0.827–0.661)	0.826
**Coffee consumption (“Yes”)**	1351 (63.5)	0.064 (−0.121–0.250)	0.497
**Sweet drinks consumption (“Yes”)**	391 (18.4)	−0.108 (−0.339–0.124)	0.362
**Sweet snacks (“Yes”)**	294 (13.8)	0.053 (−0.207–0.313)	0.689

Data are presented as numbers (percentages). Reference knowledge category is “low”, and “no”. OR, odds ratio; 95% CI, 95% confidence interval. * *p* ≤ 0.05.

## Data Availability

The data are available from the corresponding author upon reasonable request.
